# YKL-40 promotes invasion and metastasis of bladder cancer by regulating epithelial mesenchymal transition

**DOI:** 10.1080/07853890.2021.1950920

**Published:** 2021-07-14

**Authors:** Hailong Hao, Huiqing Chen, Liwu Xie, Hongyu Liu

**Affiliations:** a Department of Urology, Shanxi Cancer Hospital, Affiliated Cancer Hospital of Shanxi Medical University, Taiyuan, Shanxi, China; b Department of Pathology, Shanxi Cancer Hospital, Affiliated Cancer Hospital of Shanxi Medical University, Taiyuan, Shanxi, China

**Keywords:** Bladder cancer, EMT, YKL-40, invasion, metastasis

## Abstract

**Objective:**

This study aims to explore the relevance between YKL-40 and recurrence and progression of bladder cancer, and determine whether YKL-40 can be used as a potential target in patients with bladder cancer.

**Methods:**

We analyzed the invasion and metastasis ability of BIU-87, UMUC3, J82, T24, 5637 and immortalized human bladder epithelial cells SVHUC1 by Transwell method. The YKL-40 expression levels in cell lines were analyzed by Western blot and qPCR.

**Results:**

The increase of YKL-40 level, especially in tumour group, was related to tumour pathological stage and tumour invasion and metastasis. The cell lines with YKL-40 high expression had stronger invasion and metastasis ability. Overexpression of YKL-40 in SVHUC1 with the lowest YKL-40 expression can enhance the invasion and migration of cells. In T24 cells with YKL-40 high expression, transfection of shRNA plasmid targeting YKL-40 can down regulate the invasion and migration. The expression levels of N-cadherin and Vimentin in YKL-40 overexpressed SVHUC1 cells were increased, the E-cadherin expression was decreased, the Twist, Snail and Slug expression levels were increased, but they were opposite in T24 cells with down-regulation of YKL-40 expression.

**Conclusions:**

YKL-40 promoted the migration and invasion of bladder cancer cells by up regulating the EMT gene expression. The YKL-40 expression is closely related to the invasion and migration of bladder cancer.

## Introduction

Bladder cancer has a high incidence and mortality worldwide. Nearly 350,000 new cases are diagnosed each year, and 150,000 patients die from the disease. The incidence rate is increasing year by year [1,2]. About 70% of bladder cancers were confined to mucosa (Ta or Tis) and submucosal connective tissue (T1), i.e. non muscle invasive bladder cancer (NMIBC). The remaining 30% were intramuscular invasive bladder cancer (MIBC). NMIBC was mainly treated with transurethral resection of bladder tumour (TURBT) combined with postoperative bladder perfusion chemotherapy. However, the 5-year recurrence rate is still as high as 50 − 70%, and 10 − 15% of them progress to MIBC [3]. MIBC has a high metastasis rate and mortality rate. Despite the continuous improvement of surgical techniques, even if radical cystectomy or systemic radiotherapy and chemotherapy are accepted, about 50% of patients have distant metastasis within 2 years, and the mortality rate within 5 years is as high as 60% [4]. At present, the high recurrence rate and progression rate of NMIBC are the main problems faced by clinical doctors. Early identification of high-risk groups of NMIBC progression, accurate assessment of the risk of progression, and taking effective treatment and intervention measures to delay the progression and metastasis of tumour and reduce the mortality of tumour are very significant for improving the survival rate of patients.

YKL-40 protein (coded by CHI3L1 gene) is highly expressed in a variety of malignant tumours, and its value in disease diagnosis, evaluation and prognosis judgement has attracted more and more attention. Johansen found that YKL-40 level is an independent indicator to judge the prognosis of metastatic prostate cancer after endocrine therapy [5]. Özdemir found that YKL-40 may be a predictor of tumour load and metastasis of prostate cancer [6]. It was reported that YKL-40 could promote the invasion and metastasis of NSCLC by inducing EMT [7]. The previous study also showed that the increased expression of YKL-40 could promote the invasion and migration of prostate cancer cells [8]. Study found that YKL-40 level was bound up with the occurrence and prognosis of bladder cancer [9]. However, it is not clear whether it is related to bladder cancer metastasis, MIBC occurrence and postoperative recurrence of NMIBC.

Therefore, on the one hand, this study examined whether the YKL-40 expression in serum and tissue samples of prostate cancer patients was correlated with pathological grade, clinical stage and prognosis. On the other hand, the YKL-40 expression in bladder cancer cell lines was observed to determine whether the YKL-40 expression affected the invasion and migration of tumour cells through EMT. This study aims to explore the relevance between YKL-40 and recurrence and progression of bladder cancer, and determine whether YKL-40 can be used as a potential target in patients with bladder cancer.

## Materials and methods

### Subjects

A total of 60 cases of bladder cancer diagnosed by cystoscopy (from 2017 to 2019) were selected from urology department of Shanxi Cancer Hospital in this study. They were divided into G1, G2, G3 according to who pathological classification, and Ta, T1, T2, T3, T4 according to WHO-AJCC-TNM classification. The resected cancer and normal tissues were collected. The peripheral blood of preoperative bladder cancer patients and healthy volunteers were also collected.

### Detection of YKL-40 in serum

The serum was isolated from the peripheral blood and the supernatant was used to check the YKL-40 levels with Quantikine Human Chitinase3-like1 kit according to the instruction.

### Cells

BIU-87, UMUC3, SVHUC1, J82, T24 and 5637 cell lines were obtained from ATCC. They were cultured by RPMI 1640 with FBS (10%) at 37 °C, 5% CO_2_. pEX-3-YKL-40 and pGPU6-shYKL-40 vectors were obtained from Gene Pharma Co., Ltd. They were transfected into 2 × 10^5^/ml BIU-87 and 5637 cells respectively by Lipofectamine 2000 Reagent according to the manual. pEX3 empty vector and pGPU6 empty vector were used as negative control.

### Indirect immunofluorescence

The harvested cells were fixed by 4% paraformaldehyde at RT for 15 min. They were washed for 3 times by PBS, blocked by 10% normal serum diluted with PBS for 2 h at RT. They were incubated with antibodies (1:500 E-cadherin and 1:500 Vimentin) at RT for 2 h, washed for 3 times with PBS. The Alexa fluor 488 labelled Goat anti rabbit second antibody was added into them, they were incubated at RT for 30 min. They were mounted and observed under immunofluorescence microscope.

### RNA extraction and qRT-PCR

Total RNA was extracted following the previous reports [10,11]. The mRNA expression levels were determined by SYBR Premix Kit according to the kit’s instructions. The parameters were 95 °C, 10 min, and 40 cycles of 95 °C (10 s), 60 °C(20 s) and 72 °C (20 s). Normalization of RNA was performed using GAPDH as internal control. Quantifications was performed by the2^−△△^Ct method. Primers’ sequences were listed in Table 1.

**Table 1.** Primers used in this study.

	Primer sense	Primer sequence (5′→3′)	Size of PCR products (bp)
YKL-40	F	GAAGACTCTCTTGTCTGTCGGA	108
	R	AATGGCGGTACTGACTTGATG	
E-cadherin	F	ATTTTTCCCTCGACACCCGAT	109
	R	TCCCAGGCGTAGACCAAGA	
N-cadherin	F	TTTGATGGAGGTCTCCTAACACC	120
	R	ACGTTTAACACGTTGGAAATGTG	
Vimentin	F	AGTCCACTGAGTACCGGAGAC	98
	R	CATTTCACGCATCTGGCGTTC	
Twist	F	GCCTAGAGTTGCCGACTTATG	123
	R	TGCGTTTCCTGTTAAGGTAGC	
Snail	F	TCGGAAGCCTAACTACAGCGA	140
	R	AGATGAGCATTGGCAGCGAG	
Slug	F	CGAACTGGACACACATACAGTG	87
	R	CTGAGGATCTCTGGTTGTGGT	
GAPDH	F	TGTGGGCATCAATGGATTTGG	116
	R	ACACCATGTATTCCGGGTCAAT	

### Matrigel invasion and Transwell migration assays

The cells were digested with trypsin and diluted with serum-free medium. They were inoculated into 24 well Transwell filters with or without Matrigel with 50,000 cells/well, the medium containing 10% FBS was added to the bottom and incubated for 12 h. The cells were fixed, stained with crystal violet. The penetrating cell numbers were counted with the microscope.

### Western blot

The cells were harvested and lyzed by cell lysis solution. Total proteins were extracted and concentration was determined by BCA kit. Proteins were analyzed by SDS-PAGE electrophoresis. They were electrotransferred to a PVDF membrane and rinsed for 15 min with TBS. It was blocked and appropriate primary antibodies were added into them. They were incubated overnight at 4 °C. The membrane was incubated with the secondary antibody at RT for 2 h. The bands were determined by enhanced chemiluminescence kit. Imagequant LAS4000 (GE Healthcare, Japan) was used to observe them.

### Statistical analysis

SPSS 19.0 software was used to analyze the data. The nonparametric, 2-sided Wilcoxon rank sum test was applied for paired-group comparisons. The Cox proportional hazard regression model was used for multivariable analysis. *p* < .05 was significant.

## Results

### The YKL-40 expression in bladder cancer was increased and correlated with tumour invasion

**Figure 1.**
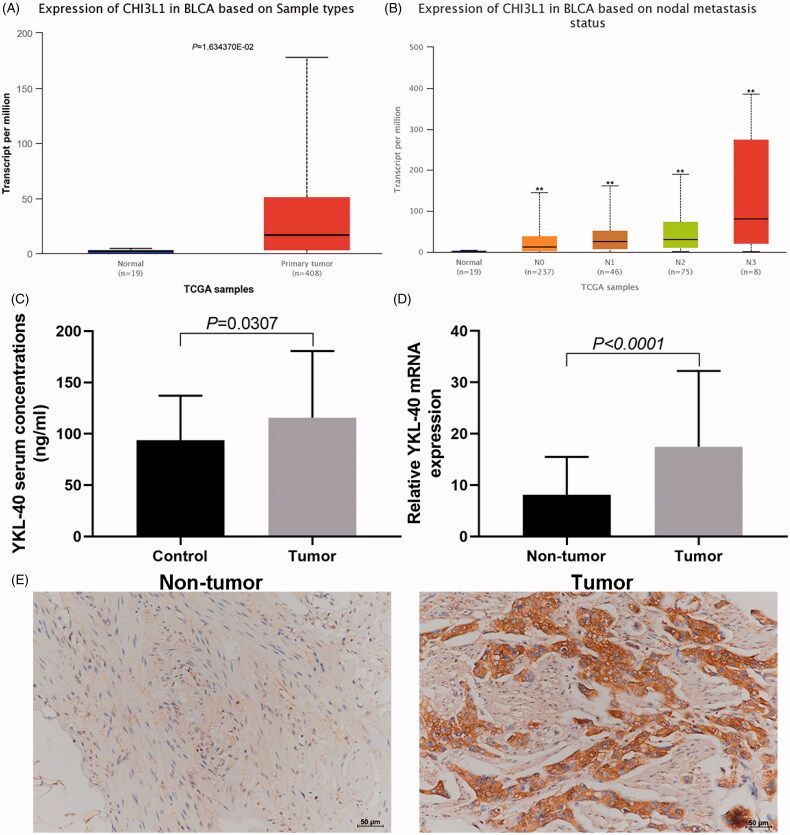
Expression of YKL-40 in serum and cancer tissues of patients with bladder cancer. (A) Online analysis using UALCAN showed that the expression level of YKL-40 coding gene CHI3L1 in bladder cancer tissues was significantly higher than that in normal; (B) The CHI3L1 expression level in bladder with different degree of lymph node metastasis (**p<.01); (C) The serum YKL-40 level in bladder cancer patients was significantly higher than that in healthy controls; (D) qPCR results of YKL-40 mRNA expression; (E): immunohistochemical detection results of YKL-40 expression.

Online analysis the data of bladder cancer in TCGA database using UALCAN (http://ualcan.path.uab.edu/) showed that the expression level of YKL-40 in bladder cancer tissues was significantly higher than that in adjacent tissues (Figure 1(A)), and the expression level increased with the increase of lymph node metastasis (Figure 1(B)). The YKL-40 serum level in patients with bladder cancer was significantly higher than that in normal volunteers (Figure 1(C)). YKL-40 levels were associated with WHO pathological stage in bladder cancer patients (Table 1). The qPCR and immunohistochemical results showed that the YKL-40 expression in cancer tissues was higher (Figure 1(D,E)), and YKL-40 mRNA level was associated with WHO pathological stage and tumour invasion (Table 2).

**Table 2.** Patients’ characteristics and YKL-40 serum and tissue levels.

Variables	Serum concentration			Tissue mRNA expression	
	*n*	Median (range)	*p*	Median (range)	*p*
Age (year)					
≤65	31	89 (27–349)	.736	2.49 (0.09–68.52)	.458
>65	29	95 (32–368)		3.25 (0.51–54.23)	
Gender					
Male	52	90 (27–368)	.762	3.41 (0.09–68.52)	.311
Female	8	94 (32–241)		2.15 (0.51–54.23)	
Stage					
Non-invasive, Ta	11	79 (27–208)	.303	1.43 (0.09–22.45)	<.001
Invasive, T1–T4	49	96 (32–368)		13.62 (0.81–68.52)	
Grade					
Low-grade, G1–G2		67 (27–199)	.041	1.31 (0.09–32.41)	<.001
High-grade, G3		103 (37–368)		14.35 (2.41–68.52)	
Lymph node					
N0/Nx		85 (27–241)	.266	2.31 (1.66–68.52)	.013
N+		95 (32–368)		7.24 (0.09–47.64)	

### The YKL-40 expression was related to invasion and migration of bladder cancer cell lines

**Figure 2.**
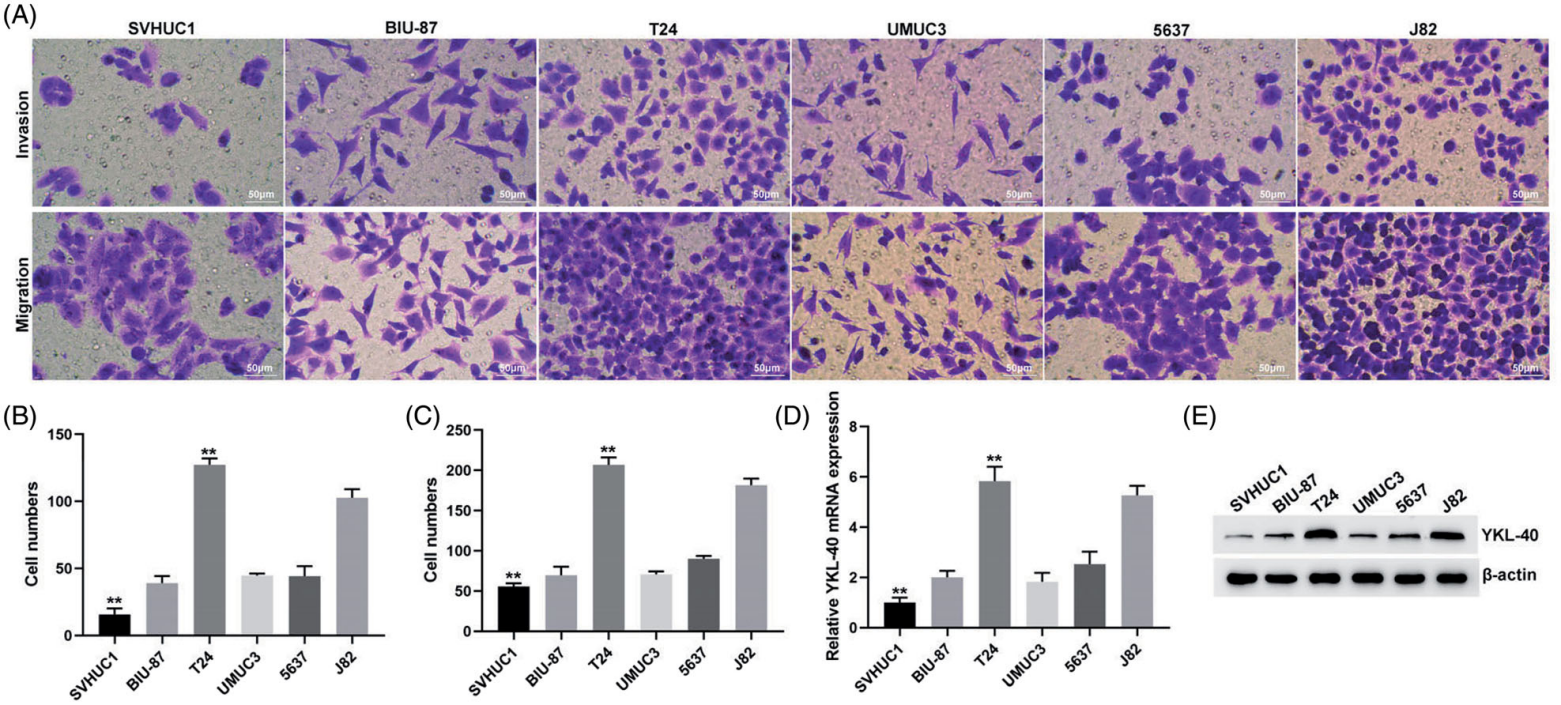
The YKL-40 expression was increased in bladder cancer cells and was related to the invasion and metastasis ability. (A) Transwell assay was used to analyze the invasion and metastasis of bladder cancer cells; (B) statistical analysis of the invasive ability of bladder cancer cells (**p<.01); (C) statistical analysis of the metastasis ability of bladder cancer cells (**p<.01); (D) qPCR results of YKL-40 mRNA expression in bladder cancer cells (**p<.01); (E) Western blotting results of YKL-40 protein expression in bladder cancer cells.

Transwell method was used to analyze the invasion and metastasis ability of BIU-87, UMUC3, J82, T24 and 5637 and SVHUC1 cells (Figure 2(A–C)). The YKL-40 expression levels in these cells were detected by Western blot and qPCR methods (Figure 2(D,E)). It was found that the cell lines with high YKL-40 expression had stronger invasion and metastasis ability.

### Changing the YKL-40 expression directly affected the ability of invasion and migration

**Figure 3.**
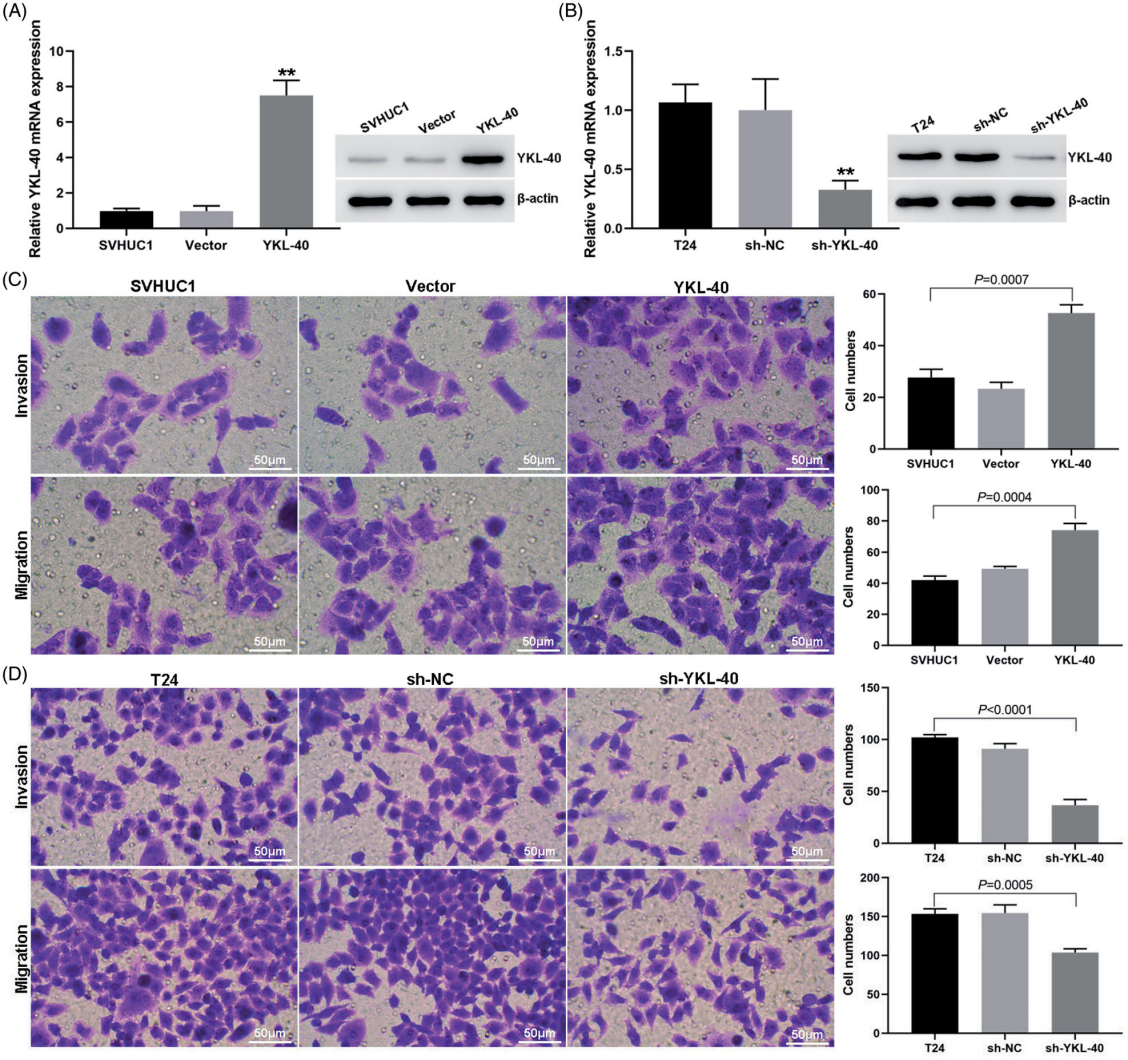
Changing the YKL-40 expression in SVHUC1 and T24 cells directly affected their ability of invasion and migration. (A) Western blot and qPCR results of YKL-40 overexpressed in SVHUC1 cells (**p<.01); (B) Western blot and qPCR results of YKL-40 shRNA transfection in T24 cells (**p<.01); (C) Transwell results of the YKL-40 overexpression effect on the invasion and metastasis of SVHUC1 cells; (D) Transwell results of the YKL-40 down regulation effect on the invasion and metastasis of T24 cells.

We selected SVHUC1 and T24 cells for subsequent experiments according to the above results. YKL-40 was overexpressed in SVHUC1 cells (Figure 3(A)) and YKL-40 expression was down regulated by shRNA in T24 cells (Figure 3(B)), respectively. Transwell analysis showed that the invasion and metastasis ability of YKL-40 overexpressed SVHUC1 cells increased significantly (Figure 3(C)), while that of YKL-40 down regulated T24 cells decreased significantly (Figure 3(D)).

### YKL-40 could directly regulate the expression of EMT related molecules

**Figure 4.**
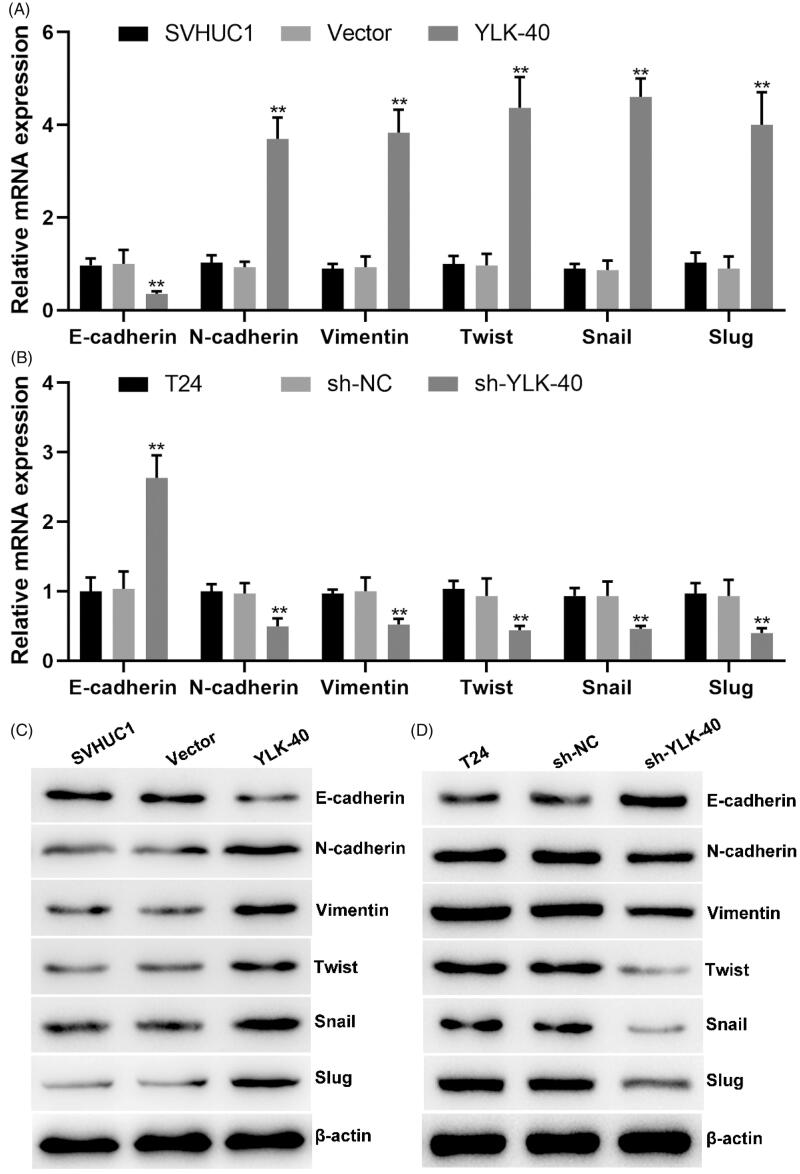
Effect of YKL-40 on EMT related gene expression. (A) The qPCR results of EMT related genes mRNA expression in SVHUC1 cells overexpressing YKL-40 (**p<.01); (B) The qPCR results of EMT related genes mRNA expression in T24 cells down regulating YKL-40 expression (**p<.01); (C) The Western blot results of EMT related genes mRNA expression in SVHUC1 cells overexpressing YKL-40; (D) The Western blot results of EMT related genes mRNA expression in T24 cells down regulating YKL-40 expression.

To analyze whether YKL-40 affects cell invasion and metastasis through EMT in bladder cancer, we detected the EMT related genes and transcription factors in SVHUC1 overexpressing YKL-40 and T24 cells down regulating YKL-40 expression, such as adhesion markers E-cadherin, Twist, Snail, Slug and N-cadherin, Vimentin. E-cadherin and vimentin were also detected by indirect immunofluorescence. The expression levels of N-cadherin and Vimentin in SVHUC1 cells over expressing YKL-40 were increased, the E-cadherin expression was decreased, the expression of Twist, Snail and Slug were increased (Figures 4(A,C) and 4). Likewise, YKL-40 knockdown T24 exhibited inhibited mesenchymal markers, and improved epithelial markers expression (Figures 4(B,D) and 4).

**Figure 5.**
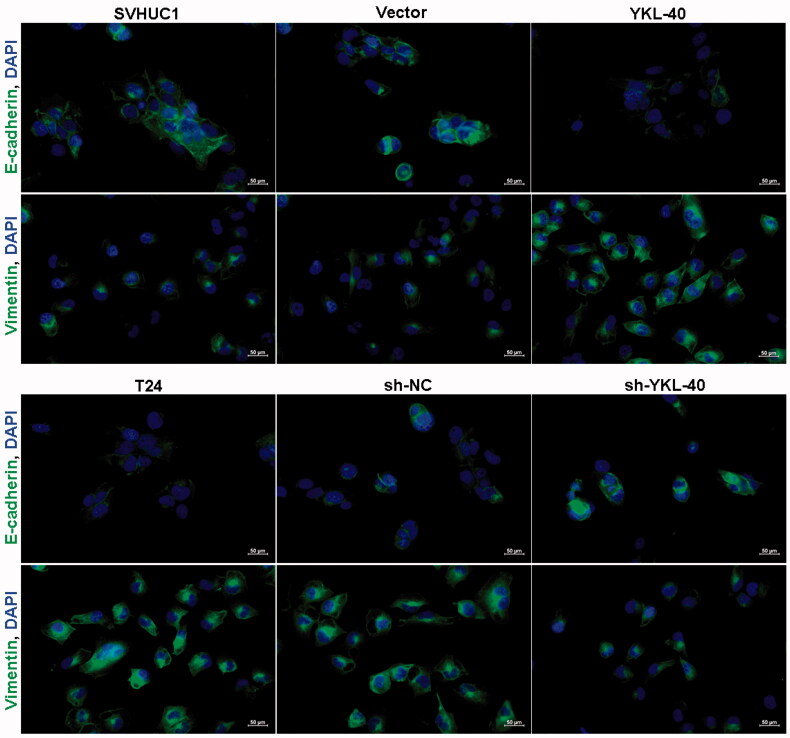
The effect of YKL-40 on the expression of E-cadherin and Vimentin was detected by indirect immunofluorescence.

## Discussion

At present, there are many treatments for bladder cancer. The choice of treatment method mainly depends on the clinical stage of the tumour. NMIBC is mainly treated with intravesical immunotherapy or chemotherapy after transurethral resection of bladder tumour. MIBC treatment requires more aggressive methods, such as radical cystectomy, radiotherapy or systemic chemotherapy [12]. In addition, bladder cancer not only has high recurrence rate and mortality rate, but also is difficult to diagnose early. Most of them are in advanced stage when diagnosed, so the chance of successful cure is greatly reduced. Therefore, it is important for bladder cancer to find suitable tumour diagnostic markers.

YKL-40 is a glycoprotein, which can participate in the process of inflammation and tissue remodelling, but its physiological function is still unclear. Many clinical studies have shown that the abnormal increased YKL-40 participates in the occurrence of tumours. The high YKL-40 level is associated with the high metastasis rate and low survival rate of a variety of human tumours [13]. However, the YKL-40 level in tumour tissues is still unclear. In present study, we found that the YKL-40 expression was significantly increased in cancer tissues, and the YKL-40 levels in serum and tissue of bladder cancer patients were related to the degree of tumour invasion. Study showed that YKL-40 participated in proliferation, survival and invasion of tumour cells in the process of tumour inflammatory microenvironment, angiogenesis and extracellular matrix remodelling [14]. YKL-40 could enhance the VEGF expression in malignant glioma cell line U87. After irradiation, U87 upregulated YKL-40 protein expression and maintained tumour cell survival by activating serine/threonine protein kinase signal and enhancing vascular endothelial function respectively [15]. When YKL-40 antibody was used to treat osteosarcoma cell line MG63 and malignant glioma cell line U87, it was observed *in vitro* that tumour vascular endothelial formation and the corresponding VEGFR2 expression were significantly decreased. *In vivo*, YKL-40 significantly inhibited angiogenesis and tumour growth, and prolonged the survival time of mice [16]. YKL-40 expressed in breast cancer cells could promote the synthesis and secretion of MMP-9 by tumour associated macrophages, thus enhancing the invasiveness of breast cancer [17,18].

In this study, we observed that the YKL-40 expression in epithelial cells was lower than bladder cancer cells, and the invasion and migration of cells with high YKL-40 expression in different bladder cancer cells were much stronger. Overexpression of YKL-40 in SVHUC1 cells could promote their invasion and migration, inhibition of YKL-40 expression by shRNA in T24 cells could inhibit their invasion and migration.

EMT may be one of the main features of poor prognosis. We found that the expression of endothelial cell marker E-cadherin was decreased in the cells with up-regulated YKL-40 expression level, while the interstitial cell markers N-cadherin and Vimentin expression levels were increased. Regulatory factors Snail, Slug, and Twist were also significantly increased. Our results clearly showed that the YKL-40 expression was related to the expression level of EMT gene. These indicated that YKL-40 promoted the bladder cancer cells’ invasion and migration by up regulating the EMT genes expression. We speculated that YKL-40 may promote EMT through PI3K/Akt pathway.

## Conclusions

In summary, our findings showed that YKL-40 was important for promoting bladder cancer metastasis. The YKL-40 expression was closely related to the invasion and migration of bladder cancer. YKL-40 promoted bladder cancer metastasis through regulating EMT genes.

## Ethical approval

This study was approved by the Ethics Committee of Shanxi Cancer Hospital. All participants signed the informed consent.

## Data Availability

The datasets during and/or analyzed during the current study are available from the corresponding author on reasonable request.

## References

[1] Mattiuzzi C, Lippi G. Current cancer epidemiology. J Epidemiol Glob Health. 2019;9(4):217–222. 31854162 10.2991/jegh.k.191008.001 PMC7310786

[2] Antoni S, Ferlay J, Soerjomataram I, et al. Bladder cancer incidence and mortality: a global overview and recent trends. Eur Urol. 2017;71(1):96–108. 27370177 10.1016/j.eururo.2016.06.010

[3] Woldu SL, Bagrodia A, Lotan Y. Guideline of guidelines: non-muscle-invasive bladder cancer. BJU Int. 2017;119(3):371–380. 28058776 10.1111/bju.13760 PMC5315602

[4] Bejrananda T, Pripatnanont C, Tanthanuch M, et al. Oncological outcomes of radical cystectomy for transitional cell carcinoma of bladder. J Med Assoc Thai. 2017;100(1):24–32. 29911376

[5] Johansen JS, Jensen BV, Roslind A, et al. Serum YKL-40, a new prognostic biomarker in cancer patients? Cancer Epidemiol Biomarkers Prev. 2006;15(2):194–202. 16492905 10.1158/1055-9965.EPI-05-0011

[6] Özdemir E, Çiçek T, Kaya MO. Association of serum YKL-40 level with tumor burden and metastatic stage of prostate cancer. Urol J. 2012;9(3):568–573. 22903479

[7] Çiledağ AA, Kabalak P, Çelik G, et al. High serum YKL-40 level is associated with poor prognosis in patients with lung cancer. Toraks. 2018;66(4):273–279. 10.5578/tt.67319 30683021

[8] Hao H, Wang L, Chen H, et al. YKL-40 promotes the migration and invasion of prostate cancer cells by regulating epithelial mesenchymal transition. Am J Transl Res. 2017;9(8):3749–3757. 28861166 PMC5575189

[9] Yan J, Shen P, Zheng J, et al. Clinical correlation between serum YKL-40 protein level and recurrence of non-muscle invasive bladder cancer. Ann Transl Med. 2015;3(22):354. 26807409 10.3978/j.issn.2305-5839.2015.11.22 PMC4701513

[10] Szarvas T, Becker M, Vom Dorp F, et al. Matrix metalloproteinase-7 as a marker of metastasis and predictor of poor survival in bladder cancer. Cancer Sci. 2010;101(5):1300–1308. 20180812 10.1111/j.1349-7006.2010.01506.x PMC11158564

[11] Tschirdewahn S, Reis H, Niedworok C, et al. Prognostic effect of serum and tissue YKL-40 levels in bladder cancer. Urol Oncol. 2014;32(5):663–669. 24814404 10.1016/j.urolonc.2014.02.004

[12] Martinez Rodriguez RH, Buisan Rueda O, Ibarz L. Bladder cancer: present and future. Med Clin. 2017; 149(10):449–455. 10.1016/j.medcli.2017.06.009 28736063

[13] Johansen JS, Jensen BV, Roslind A, et al. Is YKL-40 a new therapeutic target in cancer? Expert Opin Ther Targets. 2007;11(2):219–234. 17227236 10.1517/14728222.11.2.219

[14] Rusak A, Jabłońska K, Dzięgiel P. The role of YKL-40 in a cancerous process. Postepy Hig Med Dosw. 2016;70(0):1286–1299. 28026831

[15] Francescone RA, Scully S, Faibish M, et al. Role of YKL-40 in the angiogenesis, radioresistance, and progression of glioblastoma. J Biol Chem. 2011;286(17):15332–15343. 21385870 10.1074/jbc.M110.212514 PMC3083166

[16] Faibish M, Francescone R, Bentley B, et al. A YKL-40-neutralizing antibody blocks tumor angiogenesis and progression: a potential therapeutic agent in cancers. Mol Cancer Ther. 2011;10(5):742–751. 21357475 10.1158/1535-7163.MCT-10-0868 PMC3091949

[17] Libreros S, Garcia-Areas R, Shibata Y, et al. Induction of proinflammatory mediators by CHI3L1 is reduced by chitin treatment: decreased tumor metastasis in a breast cancer model. Int J Cancer. 2012;131(2):377–386. 21866546 10.1002/ijc.26379 PMC3288379

[18] Ku BM, Lee YK, Ryu J, et al. CHI3L1 (YKL-40) is expressed in human gliomas and regulates the invasion, growth and survival of glioma cells. Int J Cancer. 2011;128(6):1316–1326. 20506295 10.1002/ijc.25466

